# Changes in Gelation Properties of Silver Carp Myosin Treated by Combination of High Intensity Ultrasound and NaCl

**DOI:** 10.3390/foods11233830

**Published:** 2022-11-27

**Authors:** Xia Gao, Shengnan Yang, Juan You, Tao Yin, Shanbai Xiong, Ru Liu

**Affiliations:** 1 College of Food Science and Technology, Huazhong Agricultural University, Wuhan 430070, China; 2 Engineering Research Center of Green Development for Conventional Aquatic Biological Industry in the Yangtze River Economic Belt, Ministry of Education , Wuhan 430070, China; 3 National R&D Branch Center for Conventional Freshwater Fish Processing (Wuhan), Wuhan 430070, China

**Keywords:** myosin, combination treatment, high intensity ultrasound, low salt, gelation property, atomic force microscope

## Abstract

The molecular behavior of myosin in a low-salt environment limited the production of surimi-based products. This study aimed to investigate the effect of the combination of high intensity ultrasound (HIU) and NaCl (0.1, 0.3, 0.5 mol/L) on the physicochemical indexes of myosin. The changes were evaluated by solubility, ultraviolet (UV) spectroscopy, dynamic rheological properties, water holding capacity (WHC), microstructures, etc. For control samples, the gelation properties of myosin strengthened upon NaCl increasing. Combination of HIU and NaCl significantly improved the solubility of myosin, which was due to the conformational changes and the exposure of reactive groups. Meanwhile, the particle size of myosin obviously decreased when observed by atomic force microscope, which in turn promoted the stability of myosin. Furthermore, the improvement in solution behaviors of myosin treated by combination of HIU and NaCl contributed to the gelation properties as well as the formation of compact microstructures, which obtained high WHC and low cooking loss of myosin gels. In conclusion, combination of HIU and NaCl induced the unfolding of myosin with the exposure of reactive groups, consequently facilitating the formation of denser microstructures. Moreover, the biggest degree of improvement in gelation properties was observed at 0.1 mol/L NaCl combined with HIU.

## 1. Introduction

Myosin is the major functional protein responsible for the formation of surimi gels [1]. Myosin is a salt-soluble protein, which generally requires 0.47–0.68 mol/L NaCl for sufficient extraction and solubilization [2,3]. The good solubility of protein prior to heating is critical for the desirable myosin gelation [4]. During heat-induced gelling, myosin with fine dispersion first underwent conformational changes [5], facilitating the exposure of reactive groups, such as sulfhydryl groups, hydrophobic groups, etc. [6], followed by inversible protein aggregations via disulfide bonds, hydrophobic interactions and other covalent/non-covalent chemical bonds; as a result, favorable surimi gels with well textural properties formed [7,8].

Recently, the manufacture of salt-reduced surimi products has been encouraged to meet the rising demand of consumers for a healthy diet, as it is acknowledged that too much salt intake can induce health problems [9]. Nevertheless, direct reduction in salt could result in insufficient solubilization of myosin, mainly caused by formation of insoluble myosin filaments through electrostatic interactions in the rod and irregular and coarse myosin aggregations would form after heating, which is not beneficial to the ultimate formation of the desired surimi products [10]. Consequently, various strategies have been reported to produce salt-reduced surimi products with improved textural properties, such as salt substitutes, exogenous additives and alternative novel processing technologies [11,12]. Currently, high intensity ultrasound (HIU), a non-thermal processing technology, has been widely explored in the production of meat products due to its advantages of safety, easy operation and environmental friendliness [13,14]. During ultrasound, the mechanical effects (microstreaming and shear force), as well as chemical effects (free radicals), resulting from the cavitation phenomenon occurred, which were expected to disrupt protein particles [15]. Additionally, it has been reported that HIU could promote solubility as well as oxidation of reactive groups of myosin at relatively high-salt concentration (0.5 mol/L NaCl) [16,17]. Furthermore, our previous research proved that HIU improved the textural properties of sliver carp surimi with low-salt content (1% NaCl) [18]. It was speculated that this was closely related to the changes in the solubility of myosin in a low-salt environment combined with HIU. Present studies mostly focus on the effect of HIU on the physicochemical properties of myosin solutions [3] or gel properties of porcine myosin gels with 0.3 mol/L NaCl [19]. However, the changes in molecular behavior as well as the subsequent gelation properties of fish myosin treated by combination of HIU and NaCl (especially low salt of 0.1 mol/L NaCl) have rarely been reported.

To this end, myosin was subjected to the combination treatment of HIU and NaCl (0.1, 0.3, 0.5 mol/L). The solubility and conformational changes in myosin were characterized by turbidity, ultraviolet (UV) spectroscopy and sulfhydryl (SH) content. At the same time, the stability was assessed by multiple light scattering spectra. Moreover, the molecular behavior of myosin was observed by atomic force microscopy (AFM). Besides, dynamic rheology was used to monitor the gelation properties of myosin. On this basis, the gel properties and microstructures of myosin gels were further analyzed. The relationship between physicochemical indexes of myosin solution and gel properties of myosin gels were analyzed to elucidate the mechanism behind the improvement of gelation properties of myosin under HIU and NaCl combined treatment. The aim was to provide a data basis for the production of salt-reduced surimi gels.

## 2. Materials and Methods

### 2.1. Materials

Silver carp (*Hypophthalmichthys molitrix*), about 1.5 kg, was purchased from the local supermarket at Huazhong Agricultural University (Wuhan, Hubei, China). Trimethyl-amino-methane (Tris) and foline phenol were supplied by Sinopharm Chemical Reagent Co., Ltd. (Shanghai, China). All the reagents used in the present research were of analytical grade.

### 2.2. Extraction of Myosin

The extraction of myosin was performed according to Liu et al. [20] with some modifications. Firstly, the fresh fish meat was minced and homogenized with 10 folds of buffer A (containing 0.1 mol/L KCl, 0.2 mg/mL NaN_3_ and 20 mmol/L Tris-HCl, pH 7.5) for 2 min at 6000 rpm. After maintenance at 4 °C for 15 min, the mixture was centrifuged at 8000 rpm for 10 min to remove the supernatant. The remaining sedimentation was resuspended with 5 folds of buffer B (containing 0.45 mol/L KCl, 20 mmol/L Tris-HCl, 5 mmol/L β-Me, 0.2 mol/L Mg(CH_3_COO)_2_, 1 mmol/L EGTA, pH 6.8). Besides, ATP-Na_2_ was added to the final concentration of 5 mmol/L. Then, the mixture was incubated at 4 °C for 1 h, followed by centrifuging at 10,000 rpm for 10 min. The supernatant was retained and diluted with 6 folds of KHCO_3_ solution (1 mmol/L), followed by maintenance at 4 °C for 1 h and centrifuging at 12,000 rpm for 10 min. Subsequently, the sedimentation was homogenized with 2.5 folds of buffer C (containing 0.5 mol/L KCl, 5 mmol/L β-Me, pH 7.5), followed by incubating at 4 °C for 15 min and diluting with 2.5 folds of KHCO_3_ solution (1 mmol/L). At the same time, MgCl_2_ was added to the final concentration of 10 mmol/L. The homogenate was kept at 4 °C overnight, followed by centrifuging at 12,000 rpm for 15 min. The sedimentation was pure myosin. The concentration of myosin was measured by referring to Lowry’s method [21] using bovine serum protein as the standard.

### 2.3. Preparation of Myosin with Different NaCl Concentrations and HIU Treatments

For the preparation of myosin solution samples (control), the protein concentration was adjusted to 5 mg/mL using 20 mmol/L Tris-HCl buffer (containing different NaCl concentrations, pH 7.5) and the final NaCl concentration of myosin was adjusted to 0.1, 0.3 and 0.5 mol/L, respectively.

Preparation of combination-treated (HIU and NaCl) samples was performed as described by Liu et al. [17]. Briefly, 25 mL of myosin solution with different NaCl concentrations (0.1, 0.3, 0.5 mol/L) was placed in a 50 mL centrifuge tube, followed by subjecting to HIU treatment using an ultrasound processor (Ningbo Scientz Biotechnology Co., Ltd., Ningbo, China) equipped with a spherical probe (0.6 cm of diameter). During HIU, the whole sample system was surrounded with ice to prevent protein from heat-induced denaturation. The HIU parameters were 44% output for 9 min at 20 kHz. The total HIU power was 250 W and the ultrasound intensity was calculated calorimetrically according to Gao et al. [22]. In the present study, the HIU intensity was 50 W/cm^2^. The samples were stored at 4 °C for further use within 2 days.

(1) $$P=\frac{m\times c_{p}}{S}\times \frac{\mathrm{dT}}{\mathrm{dt}}$$

where P represented ultrasound intensity, W/cm^2^; m indicated the sample mass, kg; c_p_ indicated the special heat, J/(kg·K); S indicated the HIU area, cm^2^; dT/dt indicated the slope of temperature changes upon time, K/s.

For the preparation of myosin gels, the protein concentration was adjusted to 50 mg/mL as described above. Samples without or with combination (HIU and NaCl) treatment were stuffed into polyvinyl chloride casings with both ends tightly sealed, followed by heating at 40 °C for 1 h and 90 °C for 0.5 h. After heating, myosin gels were transferred to cooling water for 15 min and then kept at 4 °C overnight.

### 2.4. Determination of Solubility and Turbidity

Determination of solubility was carried out according to Li et al. [23] with minor modifications. Myosin suspension (10 mL) was centrifuged at 8000 rpm for 10 min. Subsequently, the supernatant was remained and the protein concentration was determined by Lowry’s method [21]. The solubility was calculated as the ratio of protein content in the supernatant to the total protein content.

For the determination of turbidity, the protein concentration was adjusted to 1.0 mg/mL using 20 mmol/L Tris-HCl buffer with NaCl concentrations of 0.1, 0.3, nd 0.5 mol/L, respectively. The turbidity was measured by a UV2600 spectrophotometer (Shimadzu Co., Ltd., Kyoto, Japan) and was expressed as the absorption value at 320 nm.

### 2.5. Determination of UV Spectroscopy

UV spectroscopy was determined using the method reported by Wei et al. [24]. The protein concentration was adjusted to 1.0 mg/mL. Thereafter, the samples were scanned in the range of 230 to 350 nm with intervals of 1 nm using a UV2600 spectrophotometer. The corresponding buffer was set as the blank.

### 2.6. Determination of SH Content

The reactive and total SH contents were measured by referring to the Ellman method [25]. For the determination of reactive SH content, 5.5 mL of myosin suspension (1 mg/mL) was mixed with 100 μL of Ellman’s reagent (0.2 mol/L Tris-HCl buffer, containing 10 mmol/L DTNB, pH 8.0) and the mixture was then kept at 4 °C for 1 h. For the determination of total SH content, 0.5 mL of myosin suspension (1 mg/mL) was mixed with 5 mL of 0.2 mol/L Tris-HCl buffer (pH 8.0) containing 8 mol/L urea, 10 mmol/L EDTA and 2% SDS, followed by the addition of 100 μL of Ellman’s reagent. The mixture was incubated at 40 °C for 25 min. The absorbance at 412 nm was measured and the SH content was calculated as follows:

(2) $$C_{0}=\frac{A\;\times \;D}{c\times \varepsilon }$$

where C_0_ indicated the reactive or total SH content, mol/g protein; A indicated the absorbance at 412 nm; D indicated the dilute fold of samples; c indicated the protein concentration, mg/mL; ε indicated the molar extinction coefficient, 13,600 L/(mol·cm).

### 2.7. Determination of Multiple Light Scattering Spectra

Determination of multiple light scattering spectra was carried out according to Bai et al. [26] with some modifications. Firstly, 20 mL of myosin suspension was gently transferred into a cylindrical glass bottle (about 44 mm in height) with lid, followed by detecting using the Turbiscan instrument (Turbiscan Tower, Formulation, Toulouse, France). The samples were scanned every 5 min for 6 h at 4 °C. The Turbiscan stability index (TSI) was analyzed by the Tower Software (Formulation, Toulouse, France).

### 2.8. AFM Observation

The morphology of myosin samples was observed under the instructions reported by Gao et al. [27]. The protein concentration of myosin was adjusted to 20 μg/mL, then 6 μL of myosin sample was deposited onto the surface of fresh mica. Subsequently, the samples were dried on the clean bench at room temperature overnight. After that, the samples were gently washed with 6 folds of ultrapure water 5 times to remove the extra salt and unfixed protein, followed by drying overnight. Thereafter, the myosin without or with combination (HIU and NaCl) treatment was observed by AFM (Multimode 8, Bruke Co., Billerica, MA, USA) equipped with the commercial probe (Tap 150-G) under tapping mode. The corresponding images were analyzed by NanoScope Analysis Software (Bruke Co., Billerica, MA, USA).

### 2.9. Determination of Dynamic Rheological Properties

The dynamic rheological properties of myosin were measured according to Gao et al. [18] with slight modifications. Briefly, the concentration of myosin was adjusted to 40 mg/mL, followed by determination using an AR500 dynamic rheometer (TA Co., Ltd., Manchester, England) equipped with a 40 mm parallel plate (1 mm gap). Samples were sealed with paraffin to prevent evaporation and then scanned from 4 °C to 90 °C under temperature sweep mode at a rate of 2 °C/min. The storage modulus (G’) and phase angle (δ) were recorded.

### 2.10. Determination of Water Holding Capacity (WHC) and Cooking Loss

For the determination of cooking loss, myosin sample was heated in 10 mL of centrifuge tube. Before heating, the weight of myosin and tube was recorded as W_1_ and W_2_, respectively. After two-step heating, the water loss during heating was gently wiped by filter paper and the weight of myosin gels together with tube was recorded as W_3_. The cooking loss was calculated as follows:

(3) $$\mathrm{Cooking}\;\mathrm{loss}/\%=\frac{W_{1}{+W}_{2}{-W}_{3}}{W_{2}}\times 100$$

WHC was determined according to Wang et al. [28] with slight modifications. Myosin gels were cut into slices and weighed as W_4_. Then, the slice was wrapped with three layers of filter paper, followed by centrifuging at 9000 rpm for 10 min. After centrifuging, the filter paper was removed and the weight of samples was recorded as W_5_. At least four determinations were performed. WHC was calculated as follows:

(4) $$\mathrm{WHC}/\%=\frac{W_{5}}{W_{4}}\times 100$$

### 2.11. Determination of Microstructures

The observation of microstructures was performed by referring to our previous method [29]. Myosin gels were cut into small species with the dimension of 1 × 1 × 1 mm, which were subsequently fixed with 2.5% glutaraldehyde. Next, the species were dehydrated using a series of ethanol solutions with intervals of 10% and transferred to tertiary butyl alcohol for 30 min. Thereafter, the samples were dried and coated with gold, followed by observation using a scanning electron microscope (SEM, TM 3000, Hitachi Co., Tokyo, Japan) with a magnification of 10,000.

### 2.12. Statistical Analysis

Each experiment was performed at least three times unless otherwise stated and the results were expressed as mean ± standard deviation. Origin 9.0 was used to plot figures. SPSS 22.0 was employed to assess the significant difference following two-way analysis of variance (ANOVA) together with Tukey’s test. *p* < 0.05 level indicated that there was significant difference among samples.

## 3. Results and Discussion

### 3.1. Changes in Solubility and Turbidity of Myosin Treated by Combination of HIU and NaCl

**Figure 1:**
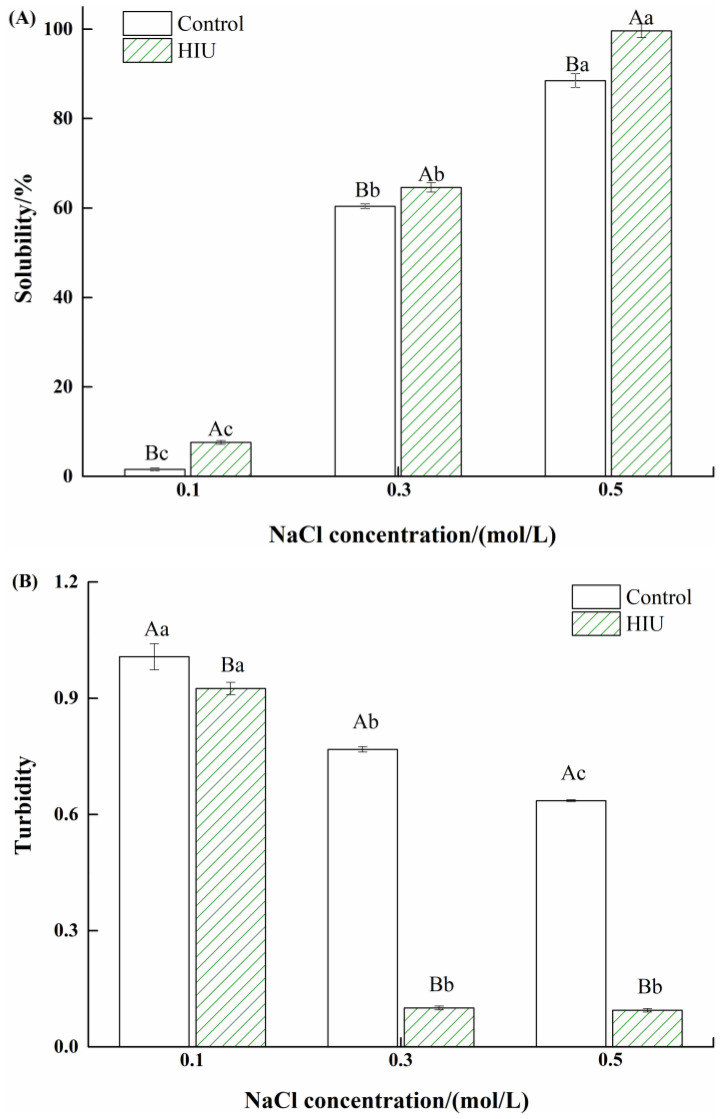
Changes in solubility (**A**) and turbidity (**B**) of myosin treated by combination of high intensity ultrasound (HIU) and NaCl. Bars indicate mean values ± standard deviations (*n* = 3). Different lowercase letters (a–c) indicate significant differences between different NaCl concentrations for control or HIU samples. Different capitals (A, B) indicate significant differences between control and HIU samples with the same NaCl concentration.

The solubility and turbidity of myosin without or with combination (HIU and NaCl) treatment are shown in Figure 1. It could be observed from Figure 1A that, for myosin treated solely by NaCl (control), the solubility was very low and only obtained 1% at 0.1 mol/L NaCl. The solubility gradually increased along with increase in the NaCl concentration and obtained 82% at 0.5 mol/L NaCl, which was nearly 50 fold that at 0.1 mol/L NaCl. Generally, myosin was salt-soluble protein, which easily tended to self-assemble and form a filamentous structure in the low-salt environment, thereby resulting in low solubility. With the further addition of NaCl, the increased negative charge repulsion originated from the charge shielding effect (Cl^−^ + NH_3_^+^) prevented myosin from polymerization, which improved the solubility.

For myosin treated by combination of HIU and NaCl, the solubility was significantly higher than the control (*p* < 0.05), irrespective of NaCl concentration. Furthermore, the solubility was improved by 30 fold at 0.1 mol/L NaCl, however, the changes were less dramatic at 0.3 or 0.5 mol/L NaCl (*p* < 0.05). It was speculated that the cavitation effect as well as the intense mechanical force induced by HIU could destroy the filamentous structure formed in the low-salt environment, which undoubtedly reduced the particle size and increased the ability of particle–water interactions, thereby promoting the solubility. Besides, the solubility of those with combination treatment was near 100% at 0.5 mol/L NaCl. Brenner et al. [30] reported that myosin existed in the form of 8–20 myosin aggregates rather than monomers in the most suitable salt environment (0.5–0.6 mol/L). The results suggested that HIU disrupted the myosin aggregates and the solubility of myosin with combination treatment was further improved in a high-salt environment (0.5 mol/L NaCl).

Turbidity is usually determined to indicate the aggregation degree of myosin molecules [31]. As shown in Figure 1B, as the NaCl concentration increased, the turbidity of myosin treated solely by NaCl (control) significantly decreased (*p* < 0.05), suggesting a reduction in the aggregation degree of myosin. As mentioned above, the myosin filaments became swollen as a result of the increased charge repulsion with the addition of NaCl, which weakened the aggregation of myosin molecules, and thus decreased turbidity was observed. This was consistent with the changes in solubility (Figure 1A). For combination-treated myosin, the turbidity was slightly but significantly lower than that of the control at 0.1 mol/L NaCl (*p* < 0.05), but sharply decreased at ≥0.3 mol/L NaCl (*p* < 0.05). The difference in turbidity for combination-treated myosin might derive from the difference in the existence form of myosin with different NaCl concentrations. According to Liu et al. [17], the decreased turbidity might be associated with the reduced particle size. HIU was expected to disrupt the myosin assemblies in the low-salt environment (0.1 mol/L NaCl) [22], while HIU was able to disrupt the myosin aggregates due to shear force at ≥ 0.3 mol/L NaCl; as a result, the particles scattered light weakly. Thus, the more obvious decrease in turbidity of combination-treated myosin was observed at ≥0.3 mol/L NaCl.

### 3.2. Changes in UV Spectroscopy of Myosin Treated by Combination of HIU and NaCl

**Figure 2:**
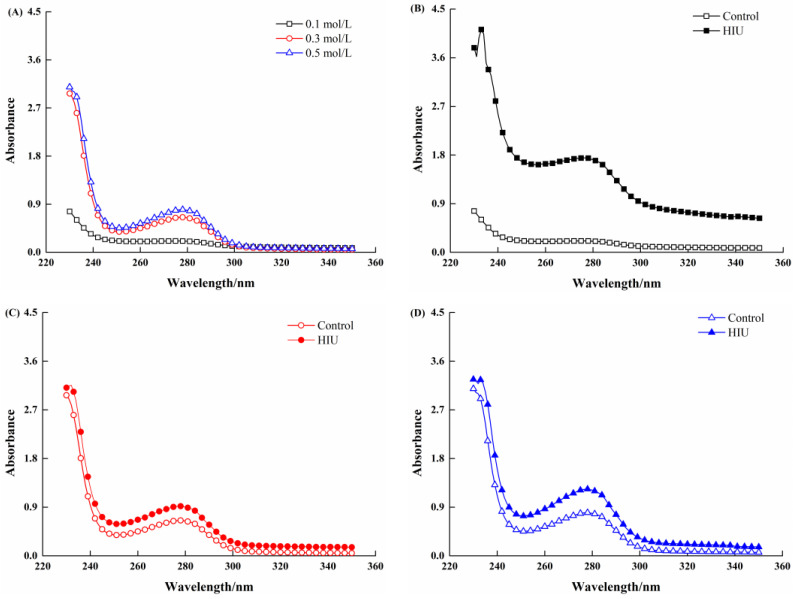
Changes in UV spectra of myosin treated by combination of HIU and NaCl. (**A**), control sample with different NaCl concentrations. (**B**), 0.1 mol/L NaCl. (**C**), 0.3 mol/L NaCl. (**D**), 0.5 mol/L NaCl.

UV spectroscopy could be used to reflect the conformational changes of myosin molecules [32]. Figure 2 shows the UV spectra of myosin without or with combination (HIU and NaCl) treatment. For all myosin samples, there was a distinct absorption peak at around 280 nm, which was attributed to the presence of aromatic amino, including tryptophan, tyrosine and phenylalanine [24]. As exhibited in Figure 2A, the absorption peak of myosin treated solely by NaCl was weak at 0.1 mol/L NaCl and the addition of NaCl increased the peak value, implying that the myosin became unfolded with the exposure of more aromatic amino residues. Additionally, the UV spectra of myosin treated by combination of HIU and NaCl were obviously higher than those of the corresponding control samples (Figure 2B–D); particularly, a larger extent in increase of absorbance was observed at 0.1 mol/L NaCl (Figure 2B). A possible reason was that the myosin filamentous structure (spontaneously formed in the low-salt environment) was largely disrupted by intense mechanical effect and thereby exposed more amino acid residues, while part of HIU energy might cause the protein degradation [18], as myosin tended to be more dispersed in the high-salt environment (0.3, 0.5 mol/L NaCl). Thus, a higher UV absorbance was obtained for myosin treated by HIU and 0.1 mol/L NaCl. These results suggested that the combination treatment further induced the exposure of aromatic amino residues originally embedded inside the myosin due to the mechanical effects, indicating the unfolding of myosin. Furthermore, this process was salt-dependent and was more prominent at low-salt concentration (0.1 mol/L NaCl). The changes in UV spectra and solubility (Figure 1A) corresponded to each other.

### 3.3. Changes in Reactive and Total SH Contents of Myosin Treated by Combination of HIU and NaCl

**Figure 3:**
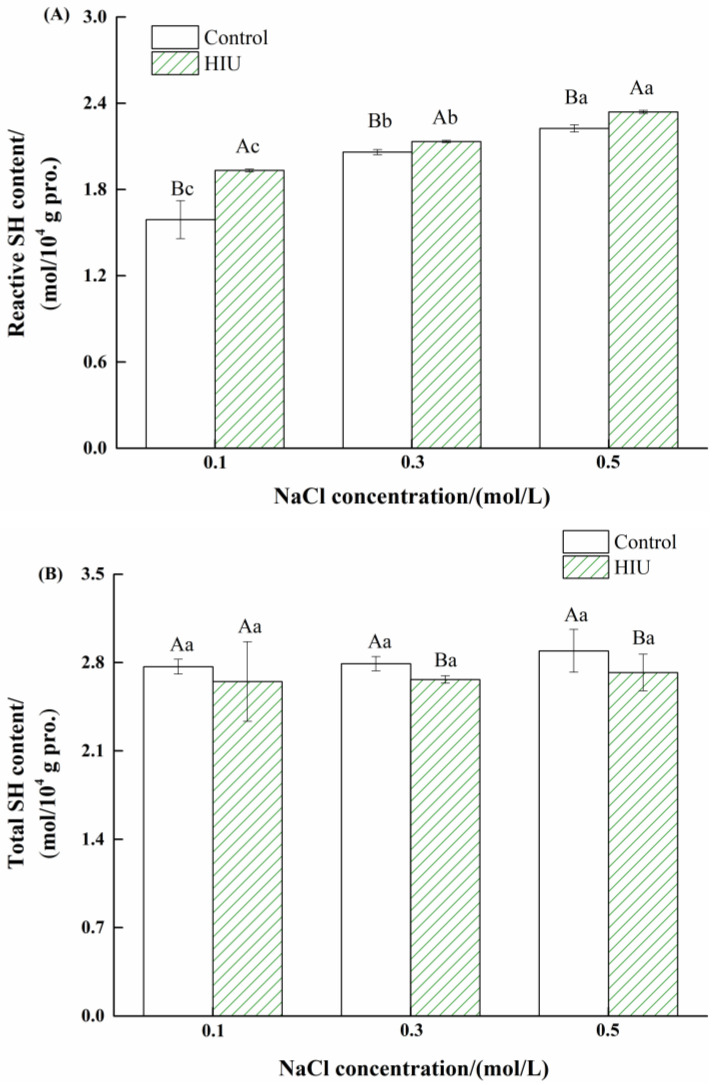
Changes in reactive (**A**) and total (**B**) sulfhydryl (SH) contents of myosin treated by combination of HIU and NaCl. Bars indicate mean values ± standard deviations (*n* = 3). Different lowercase letters (a–c) indicate significant differences between different NaCl concentrations for control or HIU samples. Different capitals (A, B) indicate significant differences between control and HIU samples with the same NaCl concentration.

The reactive and total SH contents of myosin with different treatments are illustrated in Figure 3. For control sample, the reactive SH content significantly increased upon salt increasing (*p* < 0.05) and obtained a maximum value at 0.5 mol/L NaCl (Figure 3A), indicating that the myosin became more unfolding with part of reactive groups exposed. The result was consistent with Zhang et al. [33] who reported that the rate of myosin solubilization and the subsequent exposure of SH groups was enhanced at high-salt concentration. Compared to the control, the combination of HIU and NaCl significantly improved the reactive SH content of myosin, irrespective of NaCl concentration (*p* < 0.05). Notably, the biggest improvement degree was obtained for myosin at 0.1 mol/L NaCl. The combination effect contributed to the conformational changes of myosin (Figure 2), especially at 0.1 mol/L NaCl, which probably facilitated the exposure of reactive groups.

As displayed in Figure 3B, the total SH content of untreated myosin (control) remained constant as the NaCl concentration increased. Combined with the reactive SH results, it could be concluded that the addition of NaCl was conductive to the structural changes of myosin, while rarely involving the formation of disulfide bonds. In contrast to the control, the combination treatment (HIU and NaCl) had few effects on the total SH content of myosin at 0.1 mol/L NaCl (*p* > 0.05), while significantly decreasing the total SH content of myosin at ≥0.3 mol/L NaCl (*p* < 0.05), demonstrating the conversion from -SH groups to -S-S bonds occurred at high-salt concentration (0.3, 0.5 mol/L NaCl). HIU not only generated intense mechanical effects but could also produce free radicals through the degradation of water molecules [34]. As discussed, the myosin existed as assemblies with lower solubility (Figure 1) in the low-salt environment, and it was thus speculated that the HIU energy mainly disrupted the myosin assemblies, while part of HIU energy could be saved for the oxidation of SH groups to disulfide bonds of myosin with increased solubility at high-salt concentration (0.3, 0.5 mol/L NaCl).

### 3.4. Changes in Stability of Myosin Treated by Combination of HIU and NaCl

**Figure 4:**
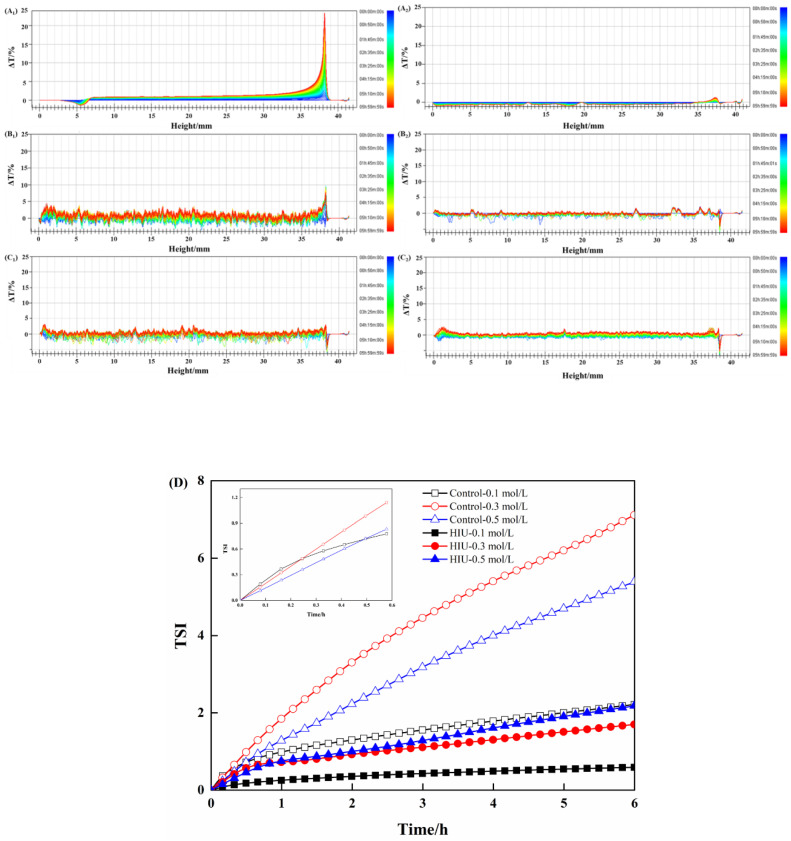
Changes in stability of myosin treated by combination of HIU and NaCl. (**A**–**C**) indicate the NaCl concentration was 0.1, 0.3 and 0.5 mol/L, respectively. 1–2 indicate control and combination treatment, respectively. (**D**) indicates the changes in Turbiscan stability index (TSI).

Turbiscan multiple light scattering can be used to detect the stability of samples by monitoring the changes in transmission light intensity during standing period [26]. The spectral lines are undoubtedly altered if the system is unstable, resulting in three typical unstable phenomena, namely, sedimentation, creaming and aggregation/flocculation [35]. The transmission light spectra of myosin without or with combination treatment are displayed in Figure 4. At the top of the myosin sample treated solely by 0.1 mol/L NaCl, the transmission light intensity increased over time, while the decreased transmission light intensity was observed at the bottom of the same sample (Figure 4A_1_), indicating that the myosin was physically unstable with particle sedimentation phenomenon occurring. This was considered to be caused by the highly ordered filamentous structure formed in the low-salt environment. As the NaCl concentration increased (Figure 4(B_1_,C_1_)), the transmission light spectra of myosin were almost steady over 6 h, reflecting that the myosin was relatively stable in the high-salt environment (0.3, 0.5 mol/L). Moreover, the more stable phenomenon was observed for myosin treated by 0.5 mol/L NaCl, as evidenced by the smallest range of transmission light spectra (Figure 4(C_1_)). The results were consistent with the increased solubility of myosin as the NaCl concentration increased (Figure 1). In addition, the combination treatment (HIU and NaCl) improved the stability of myosin, irrespective of NaCl concentration, as indicated by the smaller and smoother transmission light spectra compared to the corresponding control. It was reported by Degrand et al. [36] that the transmission light intensity depended on the particle sizes to some extent. Based on the above results, it was speculated that the combination of HIU and NaCl treatment possessed the ability to convert the myosin aggregates into smaller particles under the high shear force and turbulence derived from the instant collapse of cavitation bubbles, and thereby the improved stability of myosin was obtained.

TSI is an effective tool to estimate the stability of system: the smaller the TSI value, the more stable the system [37]. As plotted in Figure 4D, the TSI value of control samples continuously increased over time, reflecting the decreased stability of myosin. Notably, the plot of myosin treated by 0.1 mol/L NaCl differed a little from that at 0.3 or 0.5 mol/L NaCl, whose slope was high in the initial of 0.25 h but gradually slowed later. Unexpectedly, the ultimate TSI value was the smallest among control samples. Combined with the changes in transmission light spectra (Figure 4(A_1_)), a high slope probably suggested a high rate of protein sedimentation in the low-salt environment. After the protein setting down at the bottom of the bottle, it was speculated that the whole system (myosin with 0.1 mol/L NaCl) became stable. Compared to the control, the combination treatment decreased the TSI values, demonstrating that the stability of samples was largely improved. The well dispersion state would prevent protein from sedimentation due to the steric hindrance [38]. It was suggested that the myosin polymers could be disrupted by the combination effect, thus improving the dispersion and solubilization, as well as the stability.

### 3.5. Changes in Morphology of Myosin Treated by Combination of HIU and NaCl

**Figure 5:**
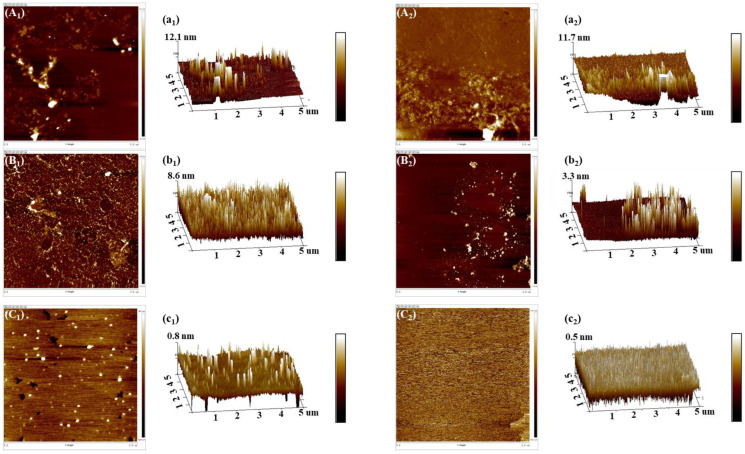
Morphology of myosin without or with combination of HIU and NaCl. (**A**–**C**) indicate the NaCl concentration was 0.1, 0.3 and 0.5 mol/L, respectively. 1–2 indicate control and combination treatment, respectively. (**a**–**c**) was the corresponding 3D image.

Figure 5 shows the surface morphology of myosin without or with combination treatment. The corresponding three-dimension structure of samples is also depicted (Figure 5(a_1_–c_2_)). For samples without combination treatment, myosin distributed unevenly on the surface of mica and assembled into filaments in the low-salt environment (0.1 mol/L NaCl) and some patches even appeared (Figure 5(A_1_)). The height of filaments ranged from 0 to 12.1 nm (Figure 5(a_1_)). This explained its poor solubility discussed in Section 3.1. It could be observed from Figure 5(B_1_) that the morphology of myosin treated by 0.3 mol/L NaCl was mainly composed of short strands, which was a little different from that of 0.1 mol/L NaCl. These strands had a smaller range of heights from 0 to 8.6 nm (Figure 5(b_1_)). As the NaCl concentration further increased to 0.5 mol/L, myosin presented in the form of oligomer rather than monosome, which overlapped on the surface of mica (Figure 5(C_1_)). Furthermore, the height of the oligomer (Figure 5(c_1_)) was apparently lower than those of samples treated by 0.1 or 0.3 mol/L NaCl. This result was in line with Brenner et al. [30] who reported that pure myosin still consisted of 8–20 monosomes under the most suitable ionic environment. In the present study, the pH of sample system was 7.5, which was higher than the isoelectric point of protein (pI = 5.5), hence, the whole sample system was dominated by many negative charges. Subsequently, the electrostatic repulsion increased while increasing the NaCl concentration, resulting in the suppression of myosin aggregation; thus, improved morphology was observed.

In contrast to the control, it could be observed that the uniform distribution of particles with obviously decreased heights formed when the sample was treated by combination of HIU and NaCl, regardless of NaCl concentration. This confirmed that the combination of HIU and NaCl disrupted the myosin assemblies into small subunits, which might relate to the rupture of non-covalent bonds of myosin aggregates. This corresponded to the improved stability of myosin after combination treatment.

### 3.6. Changes in Dynamic Rheological Properties of Myosin Treated by Combination of HIU and NaCl

**Figure 6:**
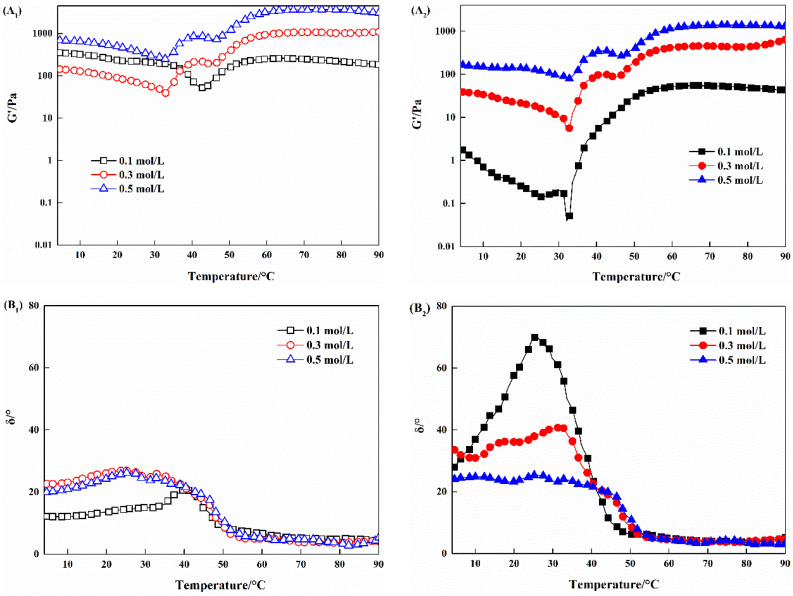
Changes in G’ (**A**) and δ (**B**) of myosin treated by combination of HIU and NaCl. 1–2 indicate control and combination treatment, respectively.

Dynamic rheology is a useful tool to evaluate the gelation properties of protein, as it recorded the changes in viscoelastic during heating [39]. Figure 6 shows the thermal gelation profiles of myosin with different treatments in terms of G’ and δ. For control samples, G’ of myosin with all NaCl concentrations initially decreased as the temperature increased from 4 °C to 32 °C. Myosin tended to aggregate via ionic bonds, hydrogen bonds, etc., at low temperature (4 °C) [40], which could be easily disrupted by high temperature, leading to the increased fluidity of samples. Thereafter, G’ of all control samples (except for samples with 0.1 mol/L NaCl) started to increase and reached the first peak at about 40 °C (setting phenomenon). This might be related to the formation of preliminary network structure cross-linked by myosin heads [20]. As a result, the samples transferred from a viscous sol to an elastic network and the increased G’, together with decreased δ, were observed. Nevertheless, myosin existed in a filamentous structure with low solubility in the low-salt environment (Figure 1), which was not beneficial for protein interactions, therefore weak setting phenomenon was observed for myosin with 0.1 mol/L NaCl. Subsequently, G’ dramatically dropped as the temperature further increased. This might derive from the disruption of preliminary structure caused by the heat-induced rupture of hydrogen bonds as well as the dissociation of myosin tails [18]. Above 50 °C, G’ continuously increased, which was considered to be owing to the formation of gel structure. Moreover, the biggest G’ value at the end of heating was obtained for myosin with 0.5 mol/L NaCl. This was consistent with its improved dispersion (Figure 1).

It is worth mentioning that the setting phenomenon occurred in myosin samples with combination of HIU and 0.1 mol/L NaCl. This might relate to the improved dispersion of myosin after combination treatment. In addition, at the end of heating, G’ of myosin with combination treatment (HIU and NaCl) was somewhat lower than the corresponding control. Combined with the similar δ of control and combination-treated samples (all lower than 5°), it was believed that the elastic gel formed for myosin after combination treatment.

### 3.7. Changes in Cooking Loss and WHC of Myosin Gels Treated by Combination of HIU and NaCl

**Figure 7:**
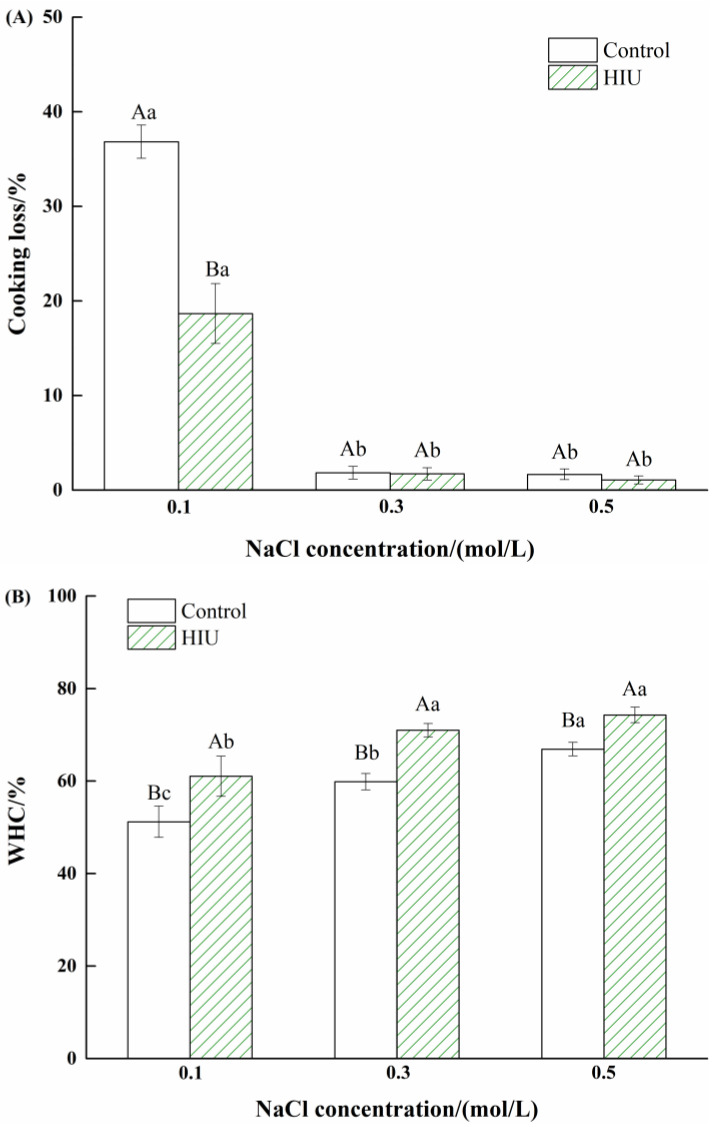
Changes in cooking loss (**A**) and water holding capacity (**B**) of myosin gels treated by combination of HIU and NaCl. Bars indicate mean values ± standard deviations (*n* = 3). Different lowercase letters (a–c) indicate significant differences between different NaCl concentrations for control or HIU samples. Different capitals (A, B) indicate significant differences between control and HIU samples with the same NaCl concentration.

The cooking loss and WHC of myosin gels without or with combination treatment are illustrated in Figure 7. The myosin gels with 0.1 mol/L NaCl alone obtained the highest cooking loss and the smallest WHC and the cooking loss was nearly 40%. Based on the solubility results (Figure 1), it was considered that the high cooking loss was caused by the smaller release of salt-soluble proteins in the low-salt environment; as a result, the protein–water interaction was weak, leading to mass water loss during cooking. As the NaCl concentration increased, the cooking loss decreased by 40% and 45%, respectively, for myosin gels treated solely by 0.3 and 0.5 mol/L NaCl, and the WHC significantly increased (*p* < 0.05), which was attributed to the increased interactions between more dispersed proteins and water molecules.

Additionally, compared to the control, the combination treatment significantly decreased the cooking loss of myosin gels at 0.1 mol/L NaCl (*p* < 0.05). Likewise, the WHC of myosin gels treated by combination treatment significantly increased compared to the control (*p* < 0.05), irrespective of NaCl concentration. As mentioned above, the increased solubility of myosin after combination of HIU and NaCl prior to heating was expected to facilitate the exposure of more water binding sites and the subsequent water–protein interactions during heating, thus improving the WHC.

### 3.8. Changes in Microstructures of Myosin Gels Treated by Combination of HIU and NaCl

**Figure 8:**
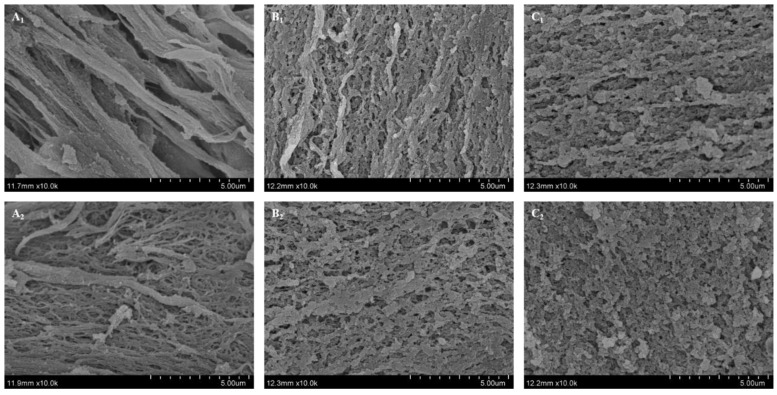
Microstructures of myosin gels without or with combination treatment. (**A**–**C**) indicate the NaCl concentration was 0.1, 0.3 and 0.5 mol/L, respectively. 1–2 indicate control and combination treatment, respectively.

The microstructures of myosin gels obtained by different treatments are presented in Figure 8. For myosin gels treated solely by 0.1 mol/L NaCl, the filamentous and coarser microstructures with large pores were observed, which explained the higher cooking loss and poor WHC (Figure 7). In the low-salt environment, myosin obtained lower solubility (Figure 1) with subsequent insufficient unfolding during gelation, leading to the disordered aggregation and irregular gel network. As NaCl concentration increased, smooth microstructures with small cavities and continuous gel matrix were obtained (Figure 8(B_1_,C_1_)). This was attribute to the increased solubility of myosin at higher NaCl concentrations (0.3, 0.5 mol/L NaCl), which facilitated the formation of more chemical bonds [18], thus forming the dense microstructures. In addition, the microstructures of myosin gels treated by combination of HIU and 0.1 mol/L NaCl became more homogenous compared to that of the control, although some filamentous aggregations remained. During HIU, part of myosin dissociated from the myosin assemblies formed in the low-salt environment (0.1 mol/L NaCl) under the cavitation effect and the protein interactions as well as chemical bonds were thus strengthened during heat-induced gelation, forming the denser gel network. Similarly, the microstructures became denser and more compact for myosin gels treated by combination treatment at 0.3 or 0.5 mol/L NaCl compared to those of the control. Notably, the biggest degree of improvement in the microstructures was observed for myosin gels with 0.1 mol/L NaCl after the combination treatment.

### 3.9. Discussion

In the present study, the changes in gelation properties of myosin treated by HIU combined with different NaCl concentrations were elucidated. For control sample (myosin solely treated by NaCl), as the NaCl concentration increased, myosin gradually dissociated and solubilized and exposed some reactive groups (i.e., amino acid residues, SH groups). During heating, dispersed myosin tended to be unfolded with further exposure of more reactive groups [41], which strengthened the subsequent protein interactions [8], contributing to the formation of compact microstructures [18]. The gelation properties of myosin were thus improved. In addition, compared to the control, combination of HIU and NaCl further improved the gelation properties of myosin, irrespective of the NaCl concentration. This was expected to be due to the cavitation effect originated from HIU, which could improve the solubility of myosin by disrupting myosin aggregates prior to heating and would in turn promote the heat-induced gelation properties. However, there was a little difference in the mechanism behind the improvement in gelation properties for myosin with solely increased-NaCl treatment and with combination treatment (the present results). It was speculated that the reason for the former (myosin with solely increased-NaCl treatment) was due to the dissociation of myosin molecules from filaments, while the reason for the latter was considered to be the disruption of myosin assemblies or aggregates prior to heating. Moreover, the biggest degree of improvement in gelation properties was observed for myosin treated by HIU and 0.1 mol/L NaCl, which further confirmed the above speculation, since the myosin assembled into filamentous structure in the low-salt environment (0.1 mol/L NaCl) and could be largely disrupted by HIU [42]. The present study might provide the theoretical basis for the production of low-salt surimi-based products.

## 4. Conclusions

For myosin treated solely by NaCl, the gelation properties were improved as the NaCl concentration gradually increased. Compared to the control, the combination of HIU and 0.1 mol/L NaCl promoted the unfolding of myosin with the exposure of more reactive groups, such as aromatic amino acid residues and SH groups, characterized by UV spectra and SH content determination. Meanwhile, the morphology observed by AFM showed that the particle size of myosin obviously decreased, which contributed to the improved solubility and stability, as supported by multiple light scattering detection. The improvements of above physicochemical indexes further contributed to the gelation properties as well as the formation of compact microstructures with smaller pores, which was beneficial for trapping more water during heat-induced gelling, thus obtaining the myosin gels with higher WHC and lower cooking loss compared to the control. In addition, the combination of HIU and NaCl also improved the gelation properties of myosin with 0.3 or 0.5 mol/L NaCl. In summary, the physicochemical and gelation properties of myosin were improved by the combination of HIU and NaCl and the largest extent of improvement was observed at low-salt environment (0.1 mol/L NaCl). This was expected to provide the data basis to guide the production of salt-reduced surimi products.

## Data Availability

The data presented in this study are available on request from the corresponding author.
